# Epigenome-wide association study of placental co-methylated regions in newborns for prenatal opioid exposure

**DOI:** 10.1093/eep/dvaf021

**Published:** 2025-09-04

**Authors:** Mandy Meijer, Chaini Konwar, Rebecca Asiimwe, Julia Maclsaac, Katia Ramadori, David Lin, Eric L Garland, Brendan Ostlund, Michael S Kobor, Sheila E Crowell, Elisabeth Conradt

**Affiliations:** Department of Medical Genetics, Faculty of Medicine, University of British Columbia, Vancouver, BC V5Z 4H4, Canada; British Columbia Children’s Hospital Research Institute, University of British Columbia, Vancouver, BC V5Z 4H4, Canada; Centre for Molecular Medicine and Therapeutics, University of British Columbia, Vancouver, BC V5Z 4H4, Canada; Department of Medical Genetics, Faculty of Medicine, University of British Columbia, Vancouver, BC V5Z 4H4, Canada; British Columbia Children’s Hospital Research Institute, University of British Columbia, Vancouver, BC V5Z 4H4, Canada; Centre for Molecular Medicine and Therapeutics, University of British Columbia, Vancouver, BC V5Z 4H4, Canada; Department of Medical Genetics, Faculty of Medicine, University of British Columbia, Vancouver, BC V5Z 4H4, Canada; British Columbia Children’s Hospital Research Institute, University of British Columbia, Vancouver, BC V5Z 4H4, Canada; Centre for Molecular Medicine and Therapeutics, University of British Columbia, Vancouver, BC V5Z 4H4, Canada; Department of Medical Genetics, Faculty of Medicine, University of British Columbia, Vancouver, BC V5Z 4H4, Canada; British Columbia Children’s Hospital Research Institute, University of British Columbia, Vancouver, BC V5Z 4H4, Canada; Centre for Molecular Medicine and Therapeutics, University of British Columbia, Vancouver, BC V5Z 4H4, Canada; Department of Medical Genetics, Faculty of Medicine, University of British Columbia, Vancouver, BC V5Z 4H4, Canada; British Columbia Children’s Hospital Research Institute, University of British Columbia, Vancouver, BC V5Z 4H4, Canada; Centre for Molecular Medicine and Therapeutics, University of British Columbia, Vancouver, BC V5Z 4H4, Canada; Department of Medical Genetics, Faculty of Medicine, University of British Columbia, Vancouver, BC V5Z 4H4, Canada; British Columbia Children’s Hospital Research Institute, University of British Columbia, Vancouver, BC V5Z 4H4, Canada; Centre for Molecular Medicine and Therapeutics, University of British Columbia, Vancouver, BC V5Z 4H4, Canada; Department of Psychiatry, University of California San Diego, San Diego, CA 92093, United States; Department of Psychology, Pennsylvania State University, State College, PA 16802, United States; Department of Medical Genetics, Faculty of Medicine, University of British Columbia, Vancouver, BC V5Z 4H4, Canada; British Columbia Children’s Hospital Research Institute, University of British Columbia, Vancouver, BC V5Z 4H4, Canada; Centre for Molecular Medicine and Therapeutics, University of British Columbia, Vancouver, BC V5Z 4H4, Canada; Edwin S. H. Leong Centre for Healthy Aging, Faculty of Medicine, University of British Columbia, Vancouver, BC V6T 1Z6, Canada; Department of Psychology, University of Oregon, Eugene, OR 97403, United States; Department of Psychology, University of Utah, Salt Lake City, UT 84112, United States

**Keywords:** opioid, DNA methylation, co-methylated region, prenatal opioid exposure, genetic predisposition, placenta, neonatal opioid withdrawal syndrome

## Abstract

The increasing incidence of opioid use during pregnancy has led to a rise in the number of infants exposed to opioids in utero. Prenatal opioid exposure may have consequences for health and (neuro)development, including neonatal opioid withdrawal syndrome (NOWS). It is unknown which infants are at greatest risk for NOWS. DNA methylation (DNAm) is an epigenetic mark reflecting both allelic variation and environmental exposures, which may provide biomarkers for prenatal opioid exposure and infant NOWS. The placenta is an accessible, biologically relevant tissue in which to directly investigate the epigenetic effects of prenatal opioid exposure. Therefore, the aims of this study were to examine whether prenatal opioid exposure is associated with differential DNAm, including epigenetic age acceleration (EAA) in the placenta. We performed an epigenome-wide association study based on co-methylated regions and single CpG sites in placental samples from in utero opioid-exposed (*n =* 19) and nonexposed infants (*n* = 143), correcting for potential confounders. We did not identify statistically significant differential DNAm profiles, but the strongest associations were found for cg06621211; cg18688392 (*ZMIZ1*, adjusted *P* = .068) and cg04460738 (*KCNMA1*, adjusted *P* = .068), although effect sizes were very small. One of these DNAm patterns (cg06621211) was in part under control of genetic variants through methylation quantitative trait loci. The involved single nucleotide polymorphism did not show significant associations in recent genome-wide association studies for phenotypes related to substance use, and the finding was not driven by potential co-occurring substance use based on sensitivity analyses. There was also no association between placental EAA and in utero opioid exposure. In conclusion, placental DNAm showed limited associations with in utero opioid exposure and NOWS diagnosis.

## Introduction

Opioids are a family of drugs that are clinically prescribed for pain relief, including anaesthesia. Opioid use, both prescribed and illicit, during pregnancy has increased substantially in recent years, with a fourfold increase among women of childbearing age between 1999 and 2014 in the USA, accompanied by a 333% increase in the number of infants exposed to opioids in utero [1], paralleling the emergence and rise of the opioid crisis [2]. Chronic, high dose use of opioids has individual health risks, including an increased mortality rate [3] and the risk for developing opioid use disorder (OUD). Additionally, prenatal opioid usage is associated with risk for foetal neurodevelopmental deficits. This class of drugs can cross the placenta and reach the foetus through receptor activation during critical windows of foetal development, thus possibly altering intracellular signalling and subsequent neurodevelopmental behaviours [4]. From a health perspective, prenatally opioid-exposed infants are at risk for neonatal opioid withdrawal syndrome (NOWS), which is a multisystem disorder specifically characterized by central nervous system irritability, gastrointestinal dysfunction, and autonomic nervous system activation [5]. NOWS-associated health outcomes include neurodevelopmental delays and possibly decreased educational attainment in childhood and adolescence [4, 6].

Epigenetics represents a key molecular process through which prenatal exposure to opioid, and co-occurring environmental factors, could affect neurodevelopmental outcomes, including NOWS [7]. One of the most stable and well-studied epigenetic modifications in human population studies is DNA methylation (DNAm), which refers to the attachment of a methyl group to a cytosine primarily in the context of a CpG dinucleotide (CpG site) [8, 9]. These DNAm patterns are highly tissue- and cell-type specific [10]. Indeed, previous studies have shown DNAm signatures to be associated with opioid exposure, both prenatally and in adulthood, in multiple tissues and organisms [11–13]. Studies in nonpregnant individuals with OUD showed increased DNAm at CpG sites in the candidate opioid receptor mu 1 (*OPRM1*) gene in blood [14, 15], lymphocytes [16], and sperm [14]—a gene known to mediate opioidergic signalling in the brain. Similarly, increases in DNAm at *OPRM1* were reported in saliva and cord blood of infants exposed to opioids and shown to be associated with the severity of NOWS [17–19]. Although effect sizes reported in these studies have been of small magnitude, typically in the range of 5% DNAm difference, these findings suggest that DNAm-based biomarkers may eventually be useful for identifying infants prenatally exposed to opioids, which subsequently might impact their health.

While DNAm in cord blood and saliva might be a relatively accessible and useful biomarker to investigate prenatal opioid exposure and subsequent health effects, the placenta is an ideal candidate tissue to explore prenatal opioid molecular signatures and its potential effects on the growing foetus as it is uniquely positioned in the maternal-foetal interface and has a crucial role in foetal development. Therefore, the placenta represents an accessible birth tissue, and foetal reprogramming via epigenetic processes in placental tissue could counterbalance the negative molecular consequences of opioids to protect the foetus. Although initially beneficial, epigenetic reprogramming could be maladaptive for development and health in the long term [20].

To date, there have only been a small number of studies on the associations between prenatal opioid exposure and DNAm in the placenta. In the context of epigenome-wide association studies (EWASs) in the placenta, one study in placental tissue samples from *White American* women identified several differentially methylated regions associated with prenatal exposures showing enrichment in genes involved in nervous system development [21]. Another study in participants of *European-American* origin identified differential methylation in opioid-exposed placentas compared to controls and demonstrated DNAm differences between opioid-exposed infants with and without NOWS [22]. However, placental cell type proportions and confounders including genetic ancestry and other substance use, that often contribute to variation in the placental methylome were not accounted for in these studies. Aside from confounders, EWAS analyses also require management of a large multiple testing burden. At present, the most commonly used platform to quantify DNAm in human populations is the Infinium HumanMethylationEPIC BeadChip (EPIC array), which measures >850 000 CpG sites throughout the genome, and therefore severe multiple test correction penalties adversely impact the ability to detect true associations. Creating co-methylated regions (CMRs) using CoMeBack takes into account the background CpG density, and therefore the high dimensionality of DNAm data can be addressed in a biologically meaningful manner [23].

CMRs and individual CpG sites are both influenced by environmental factors and genetic variants, of which the latter can be measured through methylation quantitative trait loci (mQTLs) [24]. This connection between genetic variants and DNAm should be taken into account when studying an environmental exposure (i.e. physical dependence on opioids, and subsequent opioid misuse by the mother) with a polygenic component [25]. The same is true for coexisting phenotypes, such as smoking, alcohol consumption, and psychotropic drug use [26, 27]. Therefore, it is important account for the fact that DNAm differences observed between opioid-exposed and nonexposed individuals may at least in part be due to the mQTL effects of genetic predisposition for heritable OUD rather than in utero opioid exposure [28].

In addition to DNAm association studies of single CpG sites and CMRs, the information contained in DNAm array data has been harnessed to develop biomarkers of ageing, epigenetic clocks [29–31]. A commonly derived measure from gestational epigenetic clocks is gestational epigenetic age acceleration (EAA) which suggests deviations between recorded gestational age and epigenetically-inferred gestational age likely reflective of alternations in foetal programming [32]. Gestational EAA can serve as DNAm-based proxy measures of generalized physiological disturbances, likely linked with foetal development, and associated with prenatal exposures, including but not limited to substance exposure [33–35]. Investigations testing the Developmental Origins of Health and Disease (DoHAD) theory of foetal programming are currently lacking in placenta [36].

At present, limited biomarkers are available which are associated with prenatal opioid exposure in cohorts of pregnant women receiving treatment for OUD. Identifying objective molecular markers is important for early identification of newborns at risk for NOWS and associated neurodevelopmental outcomes. Therefore, the aims of the present study were (1) to examine whether prenatal opioid exposure during OUD is associated with differential DNAm in the placenta using an EWAS approach based on both CMRs and single CpG sites in a cohort with low prevalence of polysubstance use; (2) to investigate whether the observed DNAm patterns are in part under genetic control; (3) to examine whether DNAm and genetic variants are associated with NOWS onset in infants exposed to opioids in utero; and (4) to assess whether there is a difference between EAA in infants exposed to opioids in utero compared to unexposed infants.

## Material and methods

### Study cohort and sample collection

A total of 19 pregnant women (average age 29 years, range 18–40 years) on medication-assisted treatment for OUD were recruited during their second trimester of pregnancy between April 2018 and August 2019 from speciality obstetrics/gynaecology (OB/GYN) clinics for women using substances affiliated with the University of Utah, USA. Women without opioid use during pregnancy were recruited through the Baby Affect and Behavior (BABY) study, described in detail elsewhere [37]. Even though the control samples come from a different study, data for the current study were collected in the same laboratory, and thus protocols were the same. Control samples were matched based on maternal psychopathology (emotion dysregulation), maternal race and ethnicity, infant sex, and infant gestational age (within 2 weeks). Briefly, 162 women were recruited between 2015 and 2018 during routine OB/GYN visits for a prospective study on the intergenerational transmission of emotion dysregulation. Pregnant women were oversampled for both low and high levels of emotional dysregulation to achieve a uniform distribution. Women from the BABY study were excluded if they reported any prenatal substance use or if substance use was noted in their medical record. All study participants provided written informed consent, and the University of Utah Institutional Review Board approved both studies (IRB 00107071).

For each participant, standard four-quadrant placental sampling was performed (totalling ∼1 g of tissue), and tissue 2 cm from the cord insertion site was excised within 4 h of delivery. Samples of placental parenchyma were carefully dissected by trained research assistants to be free of maternal decidua to ensure that DNA extracted from the samples was of foetal origin, all with consistent proximity to the site of umbilical cord attachment. Samples were placed immediately in RNAlater solution (Thermo Fisher Scientific, Waltham, MA, USA) and stored at 4°C. After at least 72 h, placental samples were removed from RNAlater, blotted dry, and stored in sample tubes at −80°C until used in the experiments.

### DNAm preprocessing

Genome-wide DNAm was measured in 168 placental samples using the Illumina Infinium HumanMethylationEPIC BeadChip (EPIC array), which quantifies DNAm at 866 895 CpG sites across the genome [38]. Purified DNA was quantified using an ND-1000 spectrophotometer (Marshall Scientific, Hampton, NH, USA), and samples of 1 μg of DNA were treated with bisulfite using an EZ DNA Methylation Kit (Zymo Research, Tustin, CA, USA), subjected to whole-genome amplification, and hybridised to the 850K arrays, followed by scanning with Illumina HiScan 2000 (Illumina, San Diego, CA, USA).

Raw data stored in iDAT files were read into the R programming environment with the minfi R package [39]. First, sample checks were performed using the ewastools R package [39] to (1) check if samples performed well on various control metrics (array staining, extension, hybridization, bisulphite conversion, target removal, and specificity) and (2) to confirm the concordance between self-reported sex and predicted sex inferred from DNAm intensities of the X- and Y-chromosome probes. No samples were removed based on these metrics. Next, the minfi R package was used to assess sample quality by comparing intensities of methylated and unmethylated DNA. Further, samples with a detection *P*-value in >1% of probes and samples with <3 bead count were checked as a sample quality metric. Finally, the *detectOutlier()* function in the lumi R package was utilized to identify any outliers [40]. All samples passed the aforementioned QC steps, and none were flagged for exclusion from downstream analyses.

Background correction was performed using *preprocessNoob()* in the minfi R package. Subsequently, beta mixture quantile dilation (BMIQ) normalization was performed using the wateRmelon R package to account for the probe type differences on the 850K array. As part of probe filtering step, nonautosomal XY probes were removed, as well as the 59 SNP probes present on the array to check sample identity. Next, poorly performing probes with a detection *P*-value > 0.01 in >5% of samples and bead count <3 in > 5% of samples were removed, as well as cross-hybridizing probes and probes containing SNPs based on published annotations [38], including the Illumina manifest file for the Infinium Methylation EPIC v1.0 b5. Furthermore, nonvariable probes in the placental samples were eliminated using a cutoff of 5% in DNAm values between the 10th and 90th percentiles [41]. Overall, 706 018 CpG sites in 168 individuals were retained for subsequent analyses.

Batch effects associated with the microarray chip, and chip position were corrected using *ComBat()* in the sva R package [42]. Correlation of the technical replicate pair was utilized as a quality control metric to monitor preprocessing of the data set, where the correlation coefficient improved slightly from 0.993 in raw data to 0.994 in cleaned batch-corrected data.

### Creation of CMRs

Performing association studies for all 706 018 single CpG sites suffers from a large multiple testing burden, even though not all the CpG sites in the genome are biologically independent of each other. Therefore, we accounted for correlations between adjacent CpG sites by taking the genomic background into account, and created CMRs in the measured methylome. Using the CoMeBack R package [23], we constructed CMRs with a window of 2 kb. At least four CpG sites needed to be present in the background genome, with a minimum of one present every 400 base pairs. CMRs were created when measured CpG sites had a correlation of *r* > 0.204, based on the sample size of 168 samples with available DNAm data. Composite scores for CMRs were calculated using the mean beta values of all CpG sites within the CMR

In total, we identified 101 565 CMRs, containing 340 063 probes, in our DNAm data set. A total of 324 603 single probes did not have a correlation structure with adjacent probes. Combining the CMRs and single probes resulted in a total of 426 168 CpG sites tested for association with in utero opioid exposure, thus decreasing the multiple testing burden for subsequent analyses by ∼40%.

### Cell type proportion estimation

As DNAm is cell type-specific [43] and placental tissue contains a mix of cell types, we estimated placental cell type proportions (trophoblasts, stromal cells, Hofbauer cells, endothelial cells, nucleated red blood cells, and syncytiotrophoblasts) by epigenomic deconvolution with the planet R package (Supplementary Fig. S3) [44].

### Genotyping data preprocessing

Placental samples run on the EPIC array to obtain DNAm data were also genotyped (*N*_controls_ = 151 and *N*_opiod_ = 19) using the Illumina Infinium Global Screening Array (GSAMD-24v2-0-Psych-24v1-1_20 025 904_A1) to measure SNP genotypes at 805 379 genomic loci. Using the Genotyping Module of GenomeStudio (version 2.0.4; Illumina), nonautosomal probes were removed, and GenCall scores as a measure of call rates were retrieved. Samples with a call rate ≤0.97 were excluded, or if the 10th percentile GenCall score over all SNPs for a sample was ≤0.4. This resulted in the removal of three samples. The EPIC array has 59 SNP probes that are used for quality control. Of these 59 SNP probes, 9 overlapped between the array platforms and were compared to identify sample mismatches across the arrays by performing Pearson’s correlation analysis on comparable DNAm beta values and genotyping B allele frequency values for each sample (*r*^2^ ≤ 0.9). Sex and sex aneuploidy checks for mismatches between both arrays (and against reported sex) were also performed: no mismatches were identified, and therefore no samples were removed. No samples were found to be related by both genome-wide identity-by-descent (IBD) (PI_HAT ≥ 0.1875) and SNPRelate() analyses using the method of moments (MoM) and the maximum likelihood estimation (MLE) [45, 46]. Three samples were duplicates, and samples with the lowest call rate were removed. To identify and exclude poorly performing SNP probes, newly reclustered SNPs were evaluated using Illumina’s recommended quality control metrics for probe filtering (Illumina). Probes with poor SNP calling quality (GenTrain ≤ 0.4; *n* = 4286 SNPs removed), probes that did not cluster distinctly (cluster separation score ≤ 0.45; *n* = 316 664 SNPs removed), and probes with a low percentage of samples with successful calls for the SNP in question (call frequency ≤ 0.97; *n* = 4452 SNPs removed) were removed. Next, probes with low heterozygote cluster intensities (AB R Mean ≤ 0.4), probes that were too close to a homozygote cluster (AB T Mean ≤ 0.2 or ≥ 0.8), probes with homozygote calls that clustered either too far from the axis [(AA Freq = 1 & AA T Mean ≥ 0.2) | (BB Freq == 1 & BB T Mean ≤ 0.8)], or probes that were too spread out [(AA Freq = 1 & AA T Dev ≥ 0.04) | (BB Freq == 1 & BB T Dev ≥ 0.04)], as well as probes with nearby polymorphisms [(AA.Freq == 0 & AB.T.Dev ≥ 0.5) | (BB.Freq == 0 & AB.T.Dev ≥ 0.5)], and probes that were incorrectly classified as heterozygotes [AB Freq = 0 & minor allele frequency (MAF) > 0] were excluded (*n* = 26 212 SNPs removed). Furthermore, heterozygote calls relative to Hardy–Weinberg equilibrium (HWE) expectations (Het Excess ≤ −0.3 or ≥ 0.2) or probes that had an MAF ≤ 1% were removed (*n* = 22 614 SNPs removed).

As complete or high levels of LD in genotyping data may reduce the performance of genomic prediction models [47], LD pruning was performed to retain a subset of uncorrelated SNPs. SNPs were pruned using a sliding window of 50 SNPs with the PLINK (v1.90b4)—indep command [48]. Moving the window in steps of five SNPs at a time, SNPs exceeding the variance inflation factor (VIF) threshold of 2 within each window were removed (*n* = 285 429 SNPs removed) [48]. Preselected SNPs in high-LD regions—those within the extended MHC region (8 Mb) on chromosome 6 [49] and SNPs within a 4-Mb inversion on chromosome 8 [50]—were also excluded (*n* = 4 280 SNPs removed) [51]. Finally, one sample lacking metadata was removed. Following sample and SNP filtering, 163 samples and 213 509 SNPs remained for downstream analysis.

### Genetic ancestry estimations

Self-reported ethnicity is a social construct and therefore may not accurately reflect the genetic background of individuals [52]. Several studies have reported genetic ancestry-related differences in DNAm [53, 54], which may be a confounding factor in epigenetic association studies. To account for this, we calculated genetic PCs, derived from PCA. We used the cleaned genotyping data (see *Genotyping data preprocessing* section) combined with the 2504 samples from the 1000 Genomes Project (1KGP) with multiple ancestries [55]. PCA was performed on all samples, and the first two genetic PCs were selected as covariates reflecting genetic ancestry along a continuum (Supplementary Fig. S4), which also reflected differences in the mother-reported race of the infants.

### Statistical analysis

EWASs for prenatal opioid exposure in placental tissue were performed on the combined set of CMRs and single probes without correlation structure through a robust linear regression model. We corrected for newborn sex, gestational age, birth weight, maternal income, mode of delivery, syncytiotrophoblast and trophoblast cell type proportions, the first two genetic PCs, and three technical PCs:


\begin{eqnarray*}
&& {\mathrm{DNAm}}\sim{\mathrm{opioid \, exposure}} + {\mathrm{sex}} + {\mathrm{gestational \, age}} + {\mathrm{body \, weight}} \\
&&+ \, {\mathrm{syncytiotrophoblast}} + {\mathrm{trophoblasts}} + {\mathrm{maternal \, income}} \\
&& +\, {\mathrm{mode \, of \, delivery}} + 2\,\,{\mathrm{genetic \, PCs}} + 3\,\,{\mathrm{technical \, PCs}}
\end{eqnarray*}

Missing data for the covariates were imputed using the R package missForest [55]. We calculated Benjamini–Hochberg-adjusted *P*-values. A delta beta threshold ǀ≥ 0.05ǀ was chosen as the biological cutoff, as it is likely to be greater than technical noise based on the root mean square error (RMSE) of the technical replicate pair (RMSE = 0.032).

To determine if the opioid-associated DNAm was influenced by exposure to tobacco and psychotropic drug use, contribution sensitivity analysis was performed. First, the β coefficient of opioid exposure from the base model was calculated (model 1). Then, the β coefficient of opioid exposure from the same model, adjusted for either alcohol, tobacco, or psychotropic drug use, was calculated (model 2).

Model 1: DNAm ∼ opioid exposure + sex + gestational age + body weight + syncytiotrophoblast + trophoblasts + maternal income + mode of delivery + 2 genetic PCs + 3 technical PCs
Model 2: DNAm ∼ opioid exposure + [alcohol | tobacco | psychotropic drug] + sex + gestational age + body weight + syncytiotrophoblast + trophoblasts + maternal income + mode of delivery + 2 genetic PCs + 3 technical PCs

The following contribution calculation was conducted to find the percentage of change to the effect made when incorporating a given exposure measure into the base model:


\begin{eqnarray*}
\textit{contribution} = \left( {\frac{{{{\beta }_{\textit{base}}} - {{\beta }_{adj}}}}{{{{\beta }_{\textit{base}}}}}} \right)*100.
\end{eqnarray*}

We considered a <10% contribution to be a small contribution, >10% and <25% to be a large contribution, and >25% to be a substantial contribution.

Previous candidate gene studies have focused on *OPRM1* [15, 17–19]. We extracted all single CpG sites and CMRs containing CpG sites in *OPRM1* and compared *P*-values and effect sizes with previous candidate studies.

We assessed the correlations between DNAm and expression levels of the genes annotated to the most significant CpG sites in a data set containing 48 matched placentas with available gene expression and DNAm data [56].

We determined whether the three significant DNAm associations were also associated with SNP variation. Utilizing genome-wide genotype data, associations between each CpG site and SNPs on the same chromosome within a 50-kb window were assessed using a linear model. The Benjamini–Hochberg corrected *P*-value was calculated to identify significant DNAm–SNP pairs.

Placental epigenetic age was calculated with the robust placental clock (RPC), control placental clock (CPC), and refined RPC (RRPC) [30]. Gestational EAA was calculated by using residuals extracted from a linear model by regressing epigenetic gestational age onto gestational age measured by ultrasound or last menstrual period. The difference in EAA between opioid-exposed and nonexposed infants was assessed with a linear model:


\begin{eqnarray*}
&& {\mathrm{epigenetic\, age \, acceleration}}\sim{\mathrm{opioid \, exposure}} + {\mathrm{sex}}\\
&& +\, {\mathrm{gestational \, age}} + {\mathrm{body \, weight}} + {\mathrm{syncytiotrophoblast}} \\
&& +\, {\mathrm{trophoblasts}} + {\mathrm{maternal\, income}} + {\mathrm{mode\, of\, delivery}}\\
&& + \,2\,\,{\mathrm{genetic\, PCs}}
\end{eqnarray*}

Similar to the EWAS, a sensitivity analysis was performed by adding maternal smoking, alcohol, and psychotropic medication usage to the model.

## Results

### Demographics

The study population consisted of 19 and 143 infants with and without long-term in utero opioid exposure, respectively (Table 1). While the exposed and nonexposed individuals were recruited for two different research studies, sample collection and processing were performed in the same research lab using standardized protocols, all within the same hospital. The in utero opioid-exposed infants had lower reported gestational age (*P* = .039), estimated placental syncytiotrophoblast cell type proportions (*P* = .015), and birth weight (*P* = .033), all of which are correlated with each other in our data and contribute to the DNAm data variability (Supplementary Fig. S1). Moreover, the number of vaginal births (*P* = .042) and maternal income (*P* < .001) were lower among opioid-exposed than nonexposed infants. Finally, the opioid-exposed infants had higher rates of reported exposure to maternal psychotropic drugs and tobacco (*P* < .001). Of the 19 opioid-exposed infants, 11 (58%) developed NOWS, which is diagnosed at birth.

**Table 1.** Demographic and clinical characteristics of the study cohort

Demographics	Nonexposed control group (*n* = 143) Mean (SD) or *n* (%)	Opioid-exposed group (*n* = 19) Mean (SD) or *n* (%)	*P*-value
Gestational age (weeks; reported)	39.34 (1.15)	38.83 (0.92)	.039
Syncytiotrophoblast cell type proportions	0.98 (0.06)	0.93 (0.08)	.015
Birth weight (g)	3385.21 (499.28)	3139.74 (436.30)	.033
Newborn sex (male)	72 (50.35%)	12 (63.16%)	.294
Newborn race (reported)			.103
Native American	1 (0.07%)	0 (0%)	
Asian	4 (2.80%)	1 (5.26%)	
Hawaiian or Pacific Islander	1 (0.07%)	0 (0%)	
Multiracial	30 (20.98%)	0 (0%)	
White	106 (74.13%)	15 (78.95%)	
Missing race	1 (0.07%)	3 (15.79%)	
Mother race (reported)			.336
Native American	5 (3.50%)	1 (5.26%)	
Asian	14 (9.79%)	0 (0%)	
Hawaiian or Pacific Islander	1 (0.70%)	0 (0%)	
Multiracial	35 (24.47%)	0 (0%)	
White	81 (56.64%)	6 (31.58%)	
Missing race	7 (4.90%)	12 (63.2%)	
Mother’s age at delivery (years)	29.36 (5.09)	28.16 (2.54)	.316
Mode of delivery (vaginal)			.042
Vaginal	106 (74.13%) Imputed: 107 (74.83%)	10 (52.63%) Imputed: 10 (52.63%)	
C-section	36 (25.17%) Imputed: 36 (25.17%)	9 (47.37%) Imputed: 9 (47.37%)	
Unknown	1 (0.70%) Imputed: 0 (0%)	0 (0%) Imputed: 0 (0%)	
Maternal income			<.001
<$15 000	40 (27.97%) Imputed: 41 (28.67%)	10 (52.63%) Imputed: 15 (78.95%)	
>$15 000	93 (65.03%) Imputed: 102 (71.33%)	2 (10.53%) Imputed: 3 (15.79%)	
Unknown	10 (6.99%) Imputed: 0 (0%)	7 (36.84%) Imputed: 0 (0%)	
Neonatal opioid withdrawal syndrome (NOWS)	0 (0%)	11 (57.89%)	<.001
Maternal psychotropic drug use			<.001
No	114 (79.72%) After imputation: 115 (80.42%)	8 (42.11%) After imputation: 8 (42.11%)	
Yes	26 (18.18%) After imputation: 28 (19.58%)	11 (57.89%) After imputation: 11 (57.89%)	
Unknown	3 (2.10%) After imputation: 0 (0%)	0 (0%) After imputation: 0 (0%)	
Maternal tobacco use			<.001
No	122 (85.31%) After imputation: 132 (92.31%)	5 (26.32%) After imputation: 5 (26.32%)	
Yes	2 (1.40%) After imputation: 11 (7.69%)	14 (73.68%) After imputation: 14 (73.68%)	
Unknown	19 (13.29%) After imputation: 0 (0%)	0 (0%) After imputation: 0 (0%)	
Maternal alcohol consumption			.064
No	120 (83.92%) After imputation: 136 (95.10%)	14 (73.68%) After imputation: 16 (84.21%)	
Yes	4 (2.80%) After imputation: 7 (4.90%)	2 (10.53%) After imputation: 3 (15.79%)	
Unknown	19 (13.29%) After imputation: 0 (0%)	3 (15.79%) After imputation: 0 (0%)	

### EWAS of in utero opioid exposure did not reveal differential DNAm in placenta

**Figure 1.**
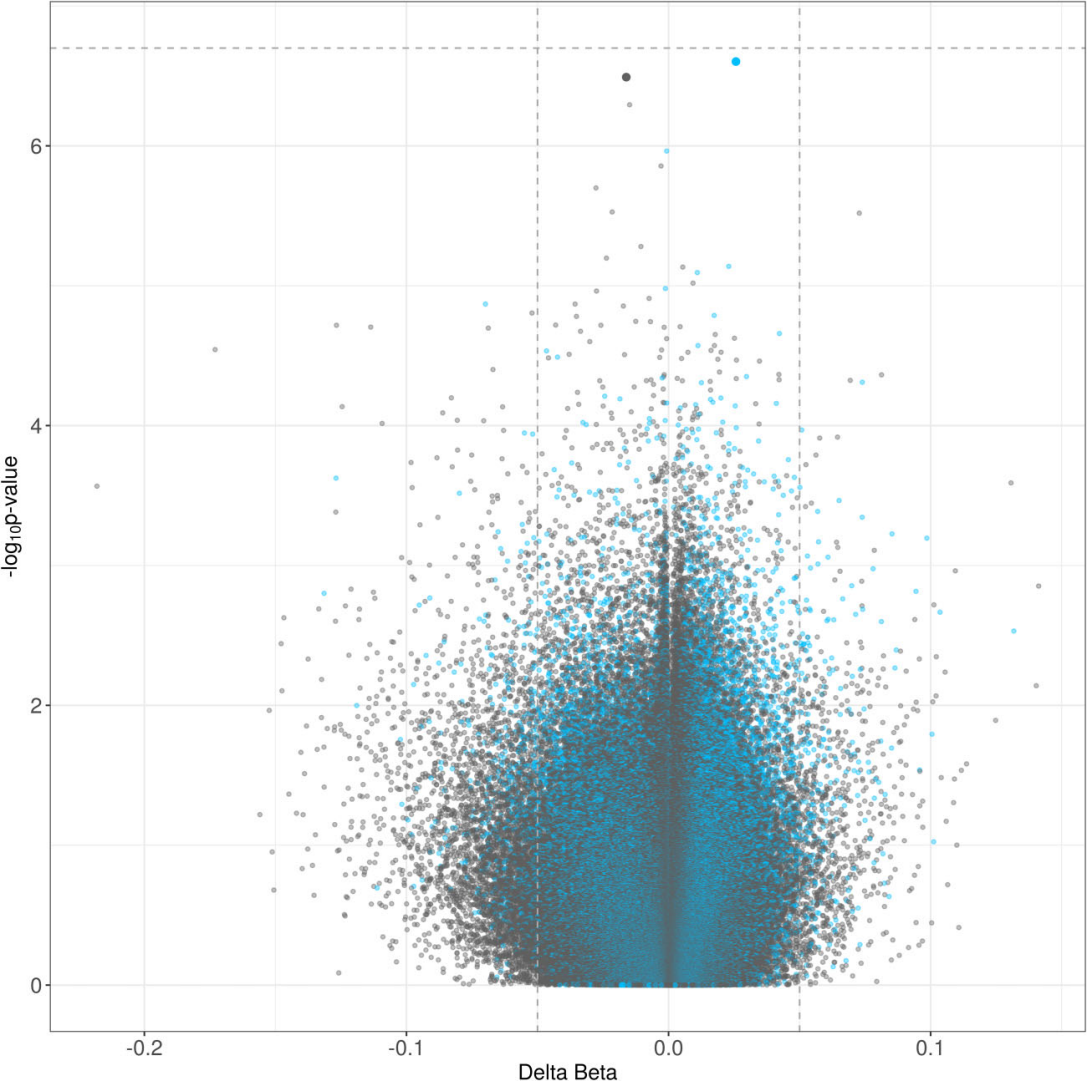
DNA methylation was not associated with in utero opioid exposure in the placenta. An association study was performed on both single CpG sites and CMRs. Individual dots in the volcano plot represent a single CpG site (grey dots) or CMR (blue dots). The *x*-axis reflects delta beta (i.e. differences in DNA methylation) between opioid-exposed and nonexposed infants. The *y*-axis reflects − log_10_(*P*-values) for the statistical model. Horizontal dashed lines represent the FDR significance threshold of 0.05. The vertical dashed line indicates the biological significant cutoff of delta beta > |0.05|.

To identify differentially methylated sites and regions associated with opioid exposure in the placenta, we conducted an EWAS on CMRs (101 565 CMRs, containing *n* = 340 063 CpG sites) and single CpG sites (324 603 CpG sites). The EWAS was corrected for sex, gestational age, body weight, placental cell type composition measured through epigenomic deconvolution, maternal income, mode of delivery, genetic PCs and technical covariates. This exploratory EWAS of in utero opioid exposure in the placenta had an inflation factor (lambda) of 1.07, and identified no significant sites after multiple testing correction [false discovery rate (FDR) < 0.05, Fig. 1, Supplementary Fig. S2]. The top-most significant findings with an FDR < 0.1 were cg04460738 (single CpG site), located in the gene body potassium calcium-activated channel subfamily M alpha 1 (*KCNMA1*) (FDR = 0.068, unadjusted *P*-value = 2.50 × 10^−7^, delta beta = 0.026), and one CMR (FDR = 0.068, unadjusted *P*-value = 3.23 × 10^−7^, delta beta = −0.016) containing two CpG sites located in the gene body of zinc finger MIZ-type containing 1 (*ZMIZ1*).

### Differential DNAm in in utero opioid associated candidate genes was not replicated in the present study

Previous candidate gene analyses showed that CpG sites in *OPRM1* are associated with in utero opioid exposure and NOWS severity in different tissues [15, 17–19, 57]. In general, these previous studies found higher DNAm levels around the *OPRM1* transcription start site (±150 bp, 2%–14% difference in DNAm) in exposed individuals compared to nonexposed individuals. To investigate whether this association was also present in our placental samples, we selected all single CpG sites and CMRs annotated to *OPRM1* in our data set (Table 2). None of the single CpG sites or CMRs in *OPRM1* in placental tissue showed a significant association with in utero opioid exposure (all, unadjusted *P* > .05). It should be noted that some of the CpG sites located in *OPRM1* reported by previous studies were not covered in our preprocessed EPIC array data set [15, 17–19, 57].

**Table 2.** Candidate CpG sites in *OPRM1*

CpG/CMR	Delta beta	Unadjusted *P*-value	Regulatory region
cg17256711	−0.016	.518	TSS1500
cg07326580; cg04912125; cg12944573	−0.010	.528	TSS1500
cg07813322	0.0005	.234	3′UTR; 5′UTR
cg00523067	−0.001	.447	3′UTR
cg19253120	0.004	.823	TSS1500

### The effects of in utero opioid exposure on placental DNAm were only partially explained by prenatal psychotropic drug use

To determine whether prenatal tobacco, alcohol, and psychotropic drug use influence the effects of in utero opioid exposure on the two topmost significant placental DNAm-associations, we performed sensitivity analyses by adding these factors as covariates to the regression model. Alcohol consumption and smoking showed minimal effect on the associations of the CMR cg06621211; cg18688392 with in utero opioid exposure: 9.73% of the initial regression coefficient was attributed to alcohol consumption or smoking (Table 3). On the contrary, smoking and alcohol consumption during pregnancy had substantial effects on the association between cg04460738 and in utero opioid exposure (<25.9%).

**Table 3.** Contribution analysis of most significant findings compared to potential confounders

	Base model		+ Smoking		+ Alcohol		+ Psychotropic drug		+ Smoking + Alcohol + Psychotropic drug	
CpG/CMR	Regression coefficient	Contribution	Regression coefficient	Contribution	Regression coefficient	Contribution	Regression coefficient	Contribution	Regression coefficient	Contribution
cg06621211; cg18688392	−0.02 37	100%	−0.021 9	7.66%	−0.02 38	0.35%	−0.02 28	3.83%	−0.02 14	9.73%
cg04460738	0.01 66	100%	0.01 23	25.9%	0.01 49	10.28%	0.01 57	5.67%	0.01 20	27.55%

### Associations of DNAm with in utero opioid exposure were under genomic control

**Figure 2.**
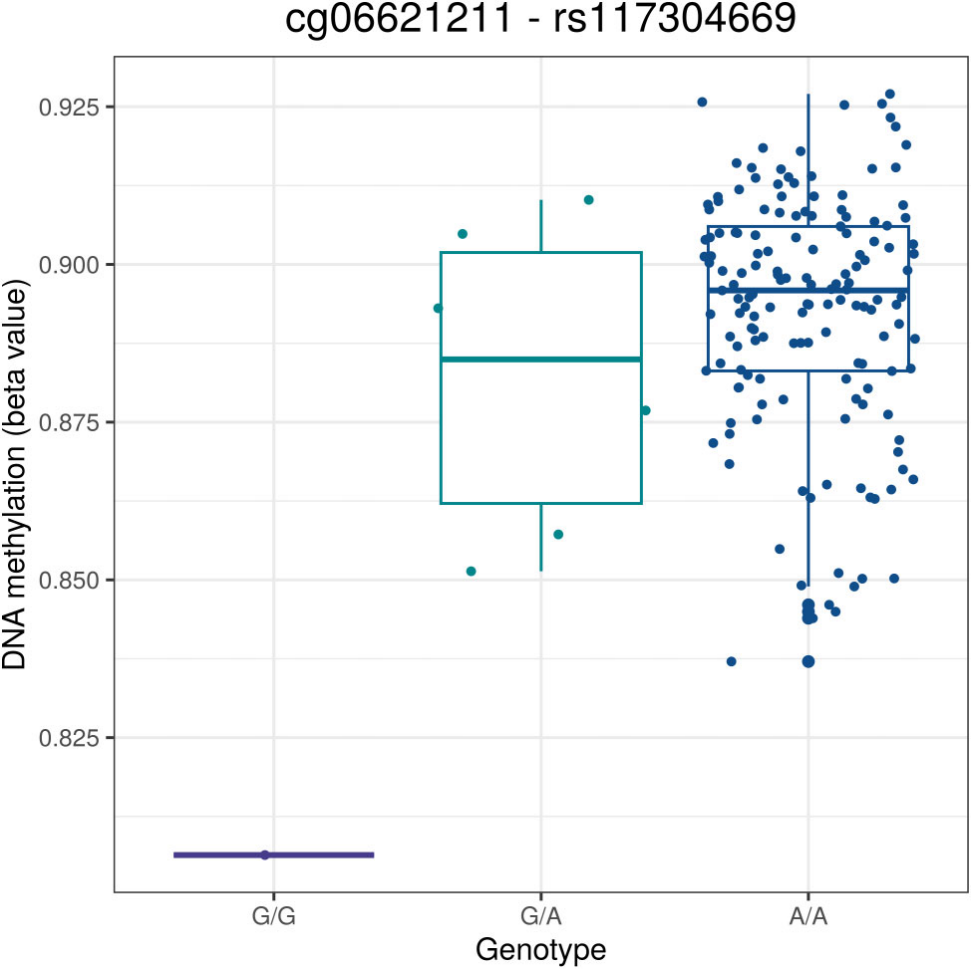
DNA methylation levels differed per genotype in mQTL pairs. DNA methylation beta values (*y*-axis) were plotted against the possible combinations of genotypes (*x*-axis) for the mQTL pairs cg06621211–rs117304669.

As DNAm levels are often influenced by genetic variants through mQTLs, we leveraged genotyping data to identify whether the two most significant DNAm-in-utero opioid exposure associations were under genomic control. One of these DNAm patterns associated with in utero opioid exposure formed an mQTL pair with a single nucleotide polymorphism (SNP) within a 50-kb window (Fig. 2). This SNP was located in zinc finger CCHC-type containing 24 (*ZCCHC24; ad hoc* Tukey *P*-value G/G—G/A and A/A < 1 × 10^−6^).

Next, we assessed whether the mQTL-SNP has been reported previously to be associated with phenotypes related to opioid exposure and substance use, as OUD has a strong genetic component, with heritability of up to 54% [58]. This SNP did not show associations with any traits in the GWAS Catalog (ebi.ac.uk/gwas, accessed on 7 October 2023). Then we looked up *P*-values and effect sizes of these SNPs in large GWASs, and none of the associations reached *P* < .05 (Supplementary Table S1). The strongest association between our mQTL-SNPs and results of existing GWASs based on *P*-values was for the number of cigarettes smoked per day [59].

### DNAm patterns were not predictive of NOWS onset

**Figure 3.**
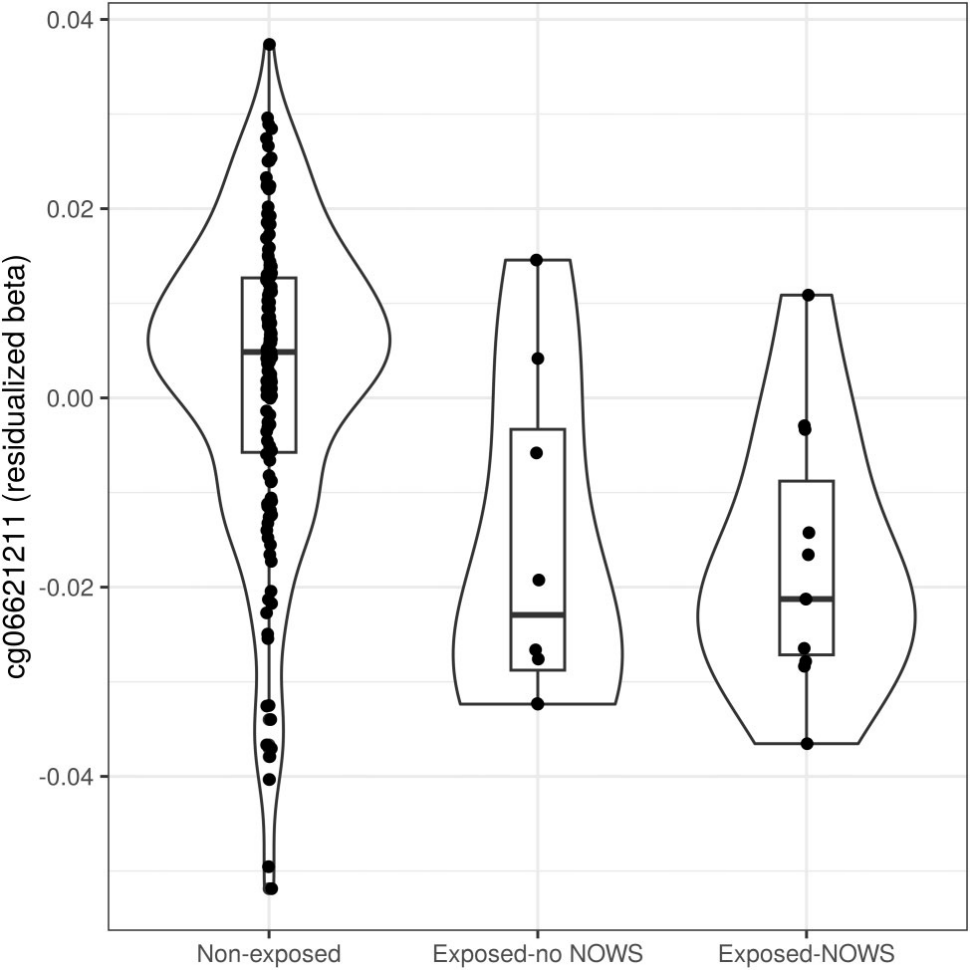
DNA methylation levels were not different in infants exposed to opioids in utero with and without NOWS. DNA methylation beta values of cg06621211 were residualized for sex and cell type proportions, and are plotted on the *y*-axis for all in utero opioid-exposed individuals without NOWS (No NOWS) and on the *x*-axis for those with NOWS.

It remains unclear why some infants develop NOWS after in utero opioid exposure, while others do not. Therefore, we investigated potential predictive DNAm biomarkers for NOWS onset. Based on the subset of 19 opioid-exposed infants, 11 of whom developed NOWS, we observed no associations between NOWS status and any relevant demographic or environmental factor: psychotropic drug use (*P* = .230), sex (*P* = .667), maternal income (*P* = .353), mode of delivery (*P* = 1.00), alcohol consumption during pregnancy (*P* = .763), smoking during pregnancy (*P* = .667), gestational age (*P* = .218), trophoblast proportion (*P* = .735), syncytiotrophoblast proportion (*P *= .761), birth weight (*P* = .916), or genetic principal component (PC) 1 (*P* = .927) or 2 (*P* = .939). Previous studies highlighted that DNAm can explain observed variation in the risk of NOWS onset and NOWS severity [17, 19, 22]. Therefore, we examined whether infants with more exaggerated DNAm patterns in the mQTL (cg06621211; cg18688392) were at higher risk of developing NOWS. Our analysis showed that DNAm values in the CMR (cg06621211; cg18688392) did not differ between individuals with and without NOWS (*P* = .89, Fig. 3).

### Neonatal opioid withdrawal syndrome was not associated with genetic variants

As the DNAm levels of cg06621211 and cg18688392 were associated with genetic variation (Fig. 2), we examined whether the risk of developing NOWS was associated with this genetic variant. We observed no difference in genotype distribution between the individuals with and without NOWS (*P* = .444, Supplementary Table S2). However, prenatal exposure to opioids was genetically associated with the mQTL-SNPs (*P* = 5.85 × 10^−4^), which can be explained by the fact that only the G/G phenotype (*n* = 1) was marginally attributable to opioid exposure (Fig. 2). Next, we examined whether DNAm associated with increased risk for NOWS is a product of a genotype-by-environment (G × E) interaction. Relevant environmental factors included psychotropic drug use, alcohol consumption, and tobacco consumption during pregnancy. None of these factors showed a G × E interaction (all, *P *> .591).

### EAA did not differ between opioid-exposed and nonexposed individuals

**Figure 4.**
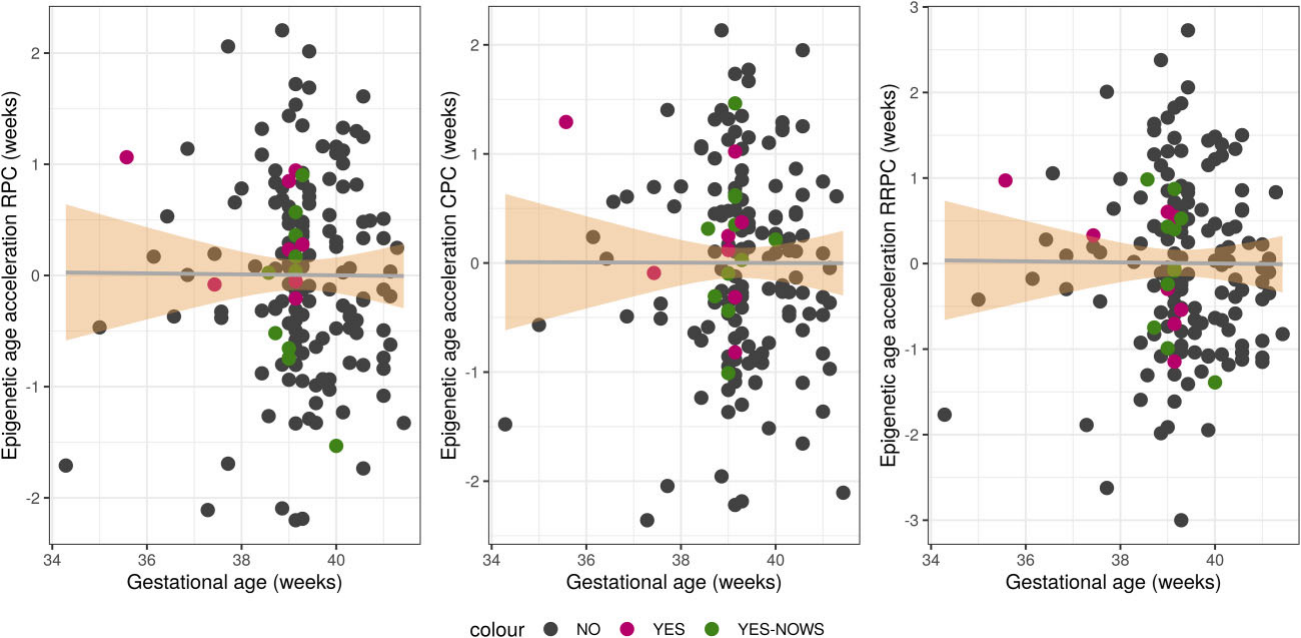
Placental epigenetic age acceleration was not associated with in utero opioid exposure. Each point represents an individual: pink, opioid-exposed infants (YES); green, opioid-exposed infants who developed neonatal opioid withdrawal syndrome (YES-NOWS); and black, nonexposed infants (NO). The *x*-axis reflects reported gestational age in weeks, and the *y*-axis reflects placental epigenetic age acceleration, measured with the robust placental clock (RPC, left), control placental clock (CPC, middle), and refined robust placental clock (RRPC, right).

Considering placental EAA as a marker of allostatic load and generalized developmental disturbances in the context of prenatal opioid exposure, we investigated differences in allostatic load between in utero opioid-exposed and nonexposed infants by calculating EAA based on three refined placental epigenetic clocks. Computed epigenetic age was correlated with reported gestational age (RPC: *r* = 0.64; CPC: *r* = 0.60; RRPC: *r* = 0.61, *P* < 2.2 × 10^−16^). However, placental EAA was not significantly different between opioid-exposed and nonexposed infants (*P* > .143) or between opioid-exposed infants with and without NOWS (*P* > .064) (Fig. 4), also not after correcting for maternal tobacco, alcohol, or psychotropic medication use (*P* > .063).

## Discussion

Long-term opioid exposure has been shown to be associated with DNAm alterations in both adults and infants in multiple tissues, but limited epigenetic biomarkers in the placenta have been reported for prenatal opioid exposure and the subsequent development of NOWS. To address this limitation, we examined whether prenatal opioid exposure was associated with differential DNAm in the placenta using region-based and single-CpG approaches in a unique study population from Utah where the overall proportion of participants consuming other substances was low. We subsequently investigated whether these DNAm patterns were in part under genetic control and whether DNAm and DNAm-related genetic variations, as determined by mQTLs, can predict NOWS onset. We did not identify any statistically significant DNAm patterns to be associated with exposure to opioids in utero, which may be reflective of our sample size, given that the magnitude of such molecular effects are subtle.

As pregnancy progresses, the number and proportion of syncytiotrophoblasts, a multinucleated epithelial cell layer covering the placental chorionic villi, increase, peaking at term. Since our opioid-exposed samples are from infants with lower gestational ages, we would expect their syncytiotrophoblast proportions to be lower compared to those of non-opioid-exposed infants. Consistent with this expectation, epigenomic deconvolution on our placental samples detected lower proportions of syncytiotrophoblast cell types in the in utero opioid-exposed infants compared to the nonexposed infants. These epithelial cells covering the placental villi facilitates oxygen and nutrient transfer between mother and foetus, primarily through passive transport based on concentration gradients and continues to grow and support the developing foetus during the pregnancy. Importantly, opioids can alter placental gene expression levels and bind to receptors on syncytiotrophoblasts [60] and have been shown to cross the placenta and disrupt in utero neurodevelopment [61]. A previous study has shown that syncytiotrophoblast aromatase immunostaining is reduced in opioid-exposed cases compared to nonexposed infants [62]. In line with the literature and our own findings that cell type proportions are highly correlated with birth weight and gestational age, the reduced syncytiotrophoblast proportions might be a response to opioid exposure, which impedes foetal growth and development.

In our EWAS using both single-CpG site and region-based approaches, the most significant differentially methylated CpG sites and regions were located in *ZMIZ1* and *KCNMA1*. The CMR we identified to be associated with opioid exposure was located in *ZMIZ1*—a transcriptional coactivator. Lower *ZMIZ1* DNAm levels have been shown to be associated with smoking status but not with nicotine dependence [63], although this CpG site (cg18688392), also identified by another EWAS on smoking [64], was not located in the current CMR. ZMIZ1 is a coactivator of the androgen receptor (AR), which has been shown to regulate transcriptional activity of the µ-opioid receptor in both rats and human neuroblastoma cell lines [65]. Therefore, the higher DNAm levels of *ZMIZ1* could be a compensatory mechanism for the high abundance of opioids reaching the placenta. Finally, KCNMA1 is involved in hormonal secretion and can be inhibited by certain opioids [66]. Differential DNAm of *KCNMA1* has been shown in both a blood-based EWAS on opioid usage among 282 users and 10 560 nonusers [67] and a placental EWAS of 19 in utero opioid-exposed infants compared to 20 nonexposed controls [21]. Finally, alterations of histone H3 lysine 27 acetylation (H3K27ac) in *KCNMA1* were detected in the brains of individuals with opioid overdose compared to individuals who died in accidents [68]. Interestingly, even though the set of DNAm sites is limited, and none of these sites have been identified to be associated with other substance use exposures, all DNAm sites identified to be differentially methylated after in utero opioid exposure are fairly proximal to the biology of opioid exposure. It should be noted that effect sizes of these DNAm sites are generally small (<5%), but nonetheless could be of high relevance in the biological understanding of the effects of in utero opioid exposure [69].

Previous candidate gene studies investigating prenatal opioid exposure identified differential DNAm in *OPRM1* [15, 17–19] in saliva and cord blood. We did not identify any association of opioid exposure with *OPRM1* DNAm in the present investigation of placental tissue. This suggests that the effects of opioid exposure on DNAm might be tissue-specific, consistent with the observation that none of the other placental EWASs on prenatal opioid exposure confirmed an association with *OPRM1* DNAm [21, 22, 57]. Alternatively, as the commercial DNAm arrays are not designed for developmental (placental) research *per se*, the arrays target different CpG sites than the candidate studies, thereby potentially missing CpG sites that may be relevant for opioid exposure in placental samples. This is consistent with the fact that some previously reported associations of *OPRM1* DNAm with opioid exposure based on candidate CpG site selection were not included on the array design [15, 17–19].

Given that opioid usage is strongly correlated with tobacco and alcohol consumption [27, 70] and thus prenatal exposure to these substances, we performed a sensitivity analysis to test whether our two most significant findings were driven by confounding substance use. As study participants lived in Utah, a state with low rates of smoking and alcohol consumption (2.8% and 10.5% in the nonexposed and exposed groups versus an average of 13.5% of mothers in the USA [71]), we had an opportunity to examine the effects of prenatal opioid exposure largely independent of polysubstance use. Our findings were consistent with a previous study of 64 placental samples, which concluded that there was no evidence that SSRIs influence placental DNAm [72].

As both DNAm levels and maternal opioid usage are influenced by genetic variance, we did not only correct our statistical analysis for genetic PCs, but we also explored the influence of genetic variants on the identified DNAm patterns and showed that one (cg06621211) DNAm pattern showed an association between DNAm levels and genetic variance. The specific mQTLs have not been observed in previous studies of mQTLs in placental samples [73, 74]. The involved SNPs were not related to a genetic predisposition for substance use, as indicated by an absence of significant associations in recent GWASs for a variety of substance use-related phenotypes (i.e. alcohol, nicotine, opioids, cannabis, over-the-counter medication). As we identified an opioid-specific DNAm signal (compared to alcohol, tobacco, and SSRIs), it is not surprising that the SNPs influencing these DNAm signals were not associated with general substance use. However, the GWASs for opioid exposure are the smallest studies of their kind, and power issues cannot rule out the possibility that these SNPs are not reflected in the inherited genetic predisposition for opioid exposure. Moreover, cross-referencing the SNPs from the mQTL analysis with existing GWAS results might not be linear given the different methodologies involved and the fact that SNPs in linkage disequilibrium (LD) can be associated in the GWASs, but not the lead-SNP

Aside from association analyses for single CpG sites and regions, we leveraged epigenetic clocks as a likely measure of allostatic load and general physiological disturbances, potentially induced by prenatal opioid exposure. These tools have been created in birth tissues, including placenta and cord blood, which are based on the DNAm levels of certain predictive CpGs that change with gestational age [30, 31]. While we observed differences in both gestational age and birth weight between opioid-exposed and non-exposed infants, no differences between epigenetic age or EAA in opioid-exposed and non-exposed infants, or infants with and without NOWS were noted, likely suggesting that opioid exposure during gestation does not impact allostatic load, proxied by EAA. Additionally, it is possible that the allostatic load induced by opioid exposure is very specific to certain CpG sites/regions rather than reflected in the CpG sites included for the epigenetic clock measures, which were developed based on only ∼3% of the total methylome. However, since previous studies have identified associations between prenatal stressors and altered EAA [75, 76], including maternal depression [77], it is also possible that we did not identify a difference between opioid-exposed and nonexposed infants because the nonexposed infants (i.e. the control group) already had a high allostatic load. Specifically, many of the mothers were selected from a study that aimed to oversample individuals with high levels of emotional dysregulation but no opioid use [78, 37]. Given its relation with chronic stress, it is plausible that emotional dysregulation would be associated with epigenetic modulation, thereby confounding our ability to assess between-group differences in allostatic load proxied by EAA.

The present study should be viewed in the context of its strengths and limitations. Owing to our small sample size, we attempted to improve our statistical power and reduce the burden of multiple testing in a biologically informed manner by first interrogating the DNAm effect of opioid exposure on 101 565 CMRs and 324 603 single CpG sites instead of the entire measured methylome at individual CpG level [23]. Furthermore, we retained CpG sites that did not fall within a CMR and conducted an association analysis in a similar manner as the region-based approach, thereby preserving as much epigenetic information of the methylome as possible and allowing an equal opportunity for all of the array CpG sites to be investigated in the context of prenatal opioid exposure. Another strength of the present study was the use of a well-characterized cohort, including demographic data of both mothers and infants, as well as information on the birth outcomes of the infants. Furthermore, pregnant individuals that did not use opioids during pregnancy came from a cohort of women with emotional dysregulation, reducing the differences in social and stressful lived experiences between the exposed and nonexposed groups [79]. It should also be acknowledged that women with OUD were on medication-assisted treatment, and therefore our findings might not be broadly generalizable for infants of opioid-using women without medication-assisted treatment, as there may be other unmeasured confounders in the latter group. Lastly, we leveraged the genotyping data on our participants to explore the effects of genetic variants in the DNAm analyses through mQTL analyses and correcting for genetic ancestry in our statistical models.

There are also some limitations to the present study that are important to highlight. The current sample size is relatively small and unbalanced, and therefore, statistical power to detect meaningful biological signals is limited, especially when taking many confounding factors and covariates into account in the statistical models used. We acknowledge that effect sizes might have been affected by this imbalance and that there might be more potential interesting unmeasured confounding factors and/or covariates that might explain DNAm differences between exposed and nonexposed individuals. However, the CMR approach helped to increase power to detect meaningful DNAm signatures, but it should be noted that the effect sizes were relatively small, and none of the DNAm associations was larger than the biological threshold set. As a result, these DNAm patterns showed overlapping values in both exposed and nonexposed individuals, as well as for the NOWS diagnosis. Thus, results presented in the current study are too limited for clinical biomarker use at this time, and it will be necessary to replicate our results in larger cohort studies, including those in populations with a diverse and under-represented racial background, to corroborate how generalizable our research findings are in these populations. Relatedly, it has to be acknowledged that multiple different opioids, including heroin and methadone, were consumed by mothers included in the current study. This could have led to an increase in heterogeneity in the DNAm profiles, and findings might not be generalizable for every type of opioid. Second, the absence of follow-up regarding developmental outcomes of infants with prenatal opioid exposure represents a limitation of this study, especially to interpret these findings in the framework of DOHaD. For other environmental exposures, including tobacco [80, 81] and alcohol exposure and foetal alcohol spectrum syndrome [82, 83], it has been shown that persistent DNAm patterns exist in other tissues. Therefore, future investigations of the stability of the biomarkers and predictive capability of (neuro)developmental outcomes as well as measures of substance usage in the offspring are required to study the role of epigenetics in long-term health outcomes of in utero opioid exposure. Finally, it is important to acknowledge the intraindividual variation observed within placental tissues. It is recommended to pool placental samples from multiple sites within the placenta to obtain representative measures of the entire placenta, and thus minimize sampling variation [84]. While only one site was sampled from the placentas in the present study, the protocol for placenta collection was the same for all samples, and placental sampling was consistently performed close to the site of umbilical cord attachment.

## Conclusions

We performed a placental EWAS of prenatal opioid exposure and did not identify DNAm signatures at the multiple-test corrected statistical threshold. The topmost significant DNAm patterns were located in *ZMIZ1* and *KCNMA1* and in part under the influence of genetic variants. Given that placental samples are readily accessible at birth, placental DNAm—which is responsive to the environment—might evolve into a future biomarker for prenatal opioid exposure detection as well as for identifying infants at risk for NOWS and for potentially predicting developmental outcomes.

## Supplementary Material

dvaf021_Supplemental_File

## Data Availability

Data are available from the corresponding author on reasonable request.
