# Supplementary material for: Epigenome-wide association study of placental co-methylated regions in newborns for prenatal opioid exposure
Source: Environ Epigenet. 2025 Sep 4;11(1):dvaf021. doi: 10.1093/eep/dvaf021 (PMC12422002; doi:10.1093/eep/dvaf021)
Supplement: dvaf021_Supplemental_File [file dvaf021_supplemental_file.docx]

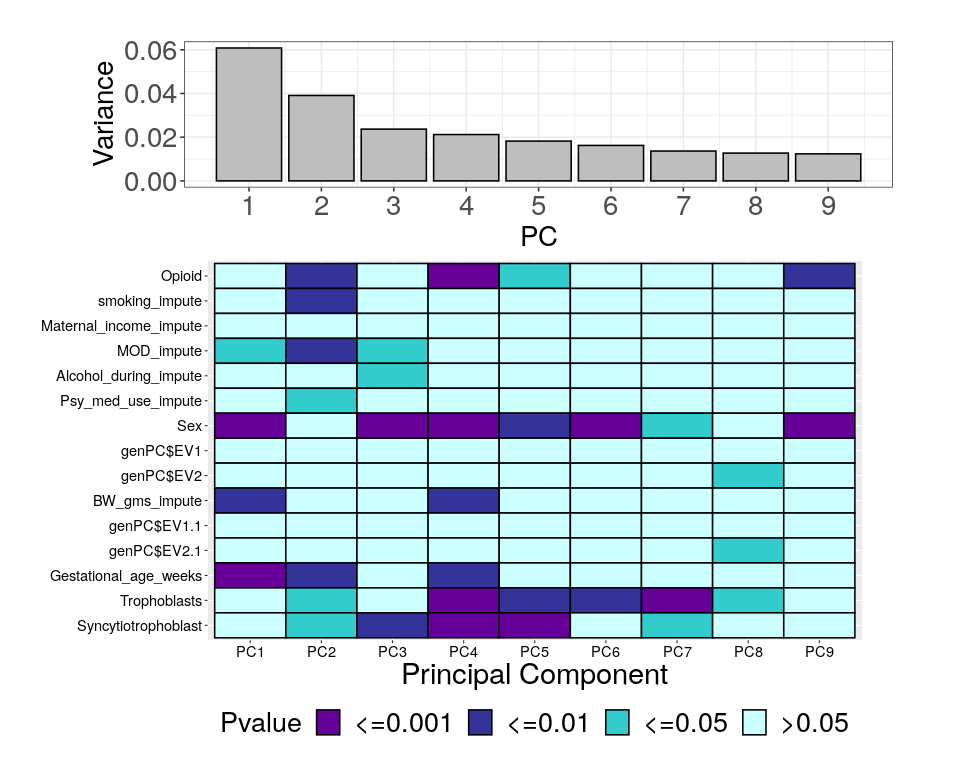
**
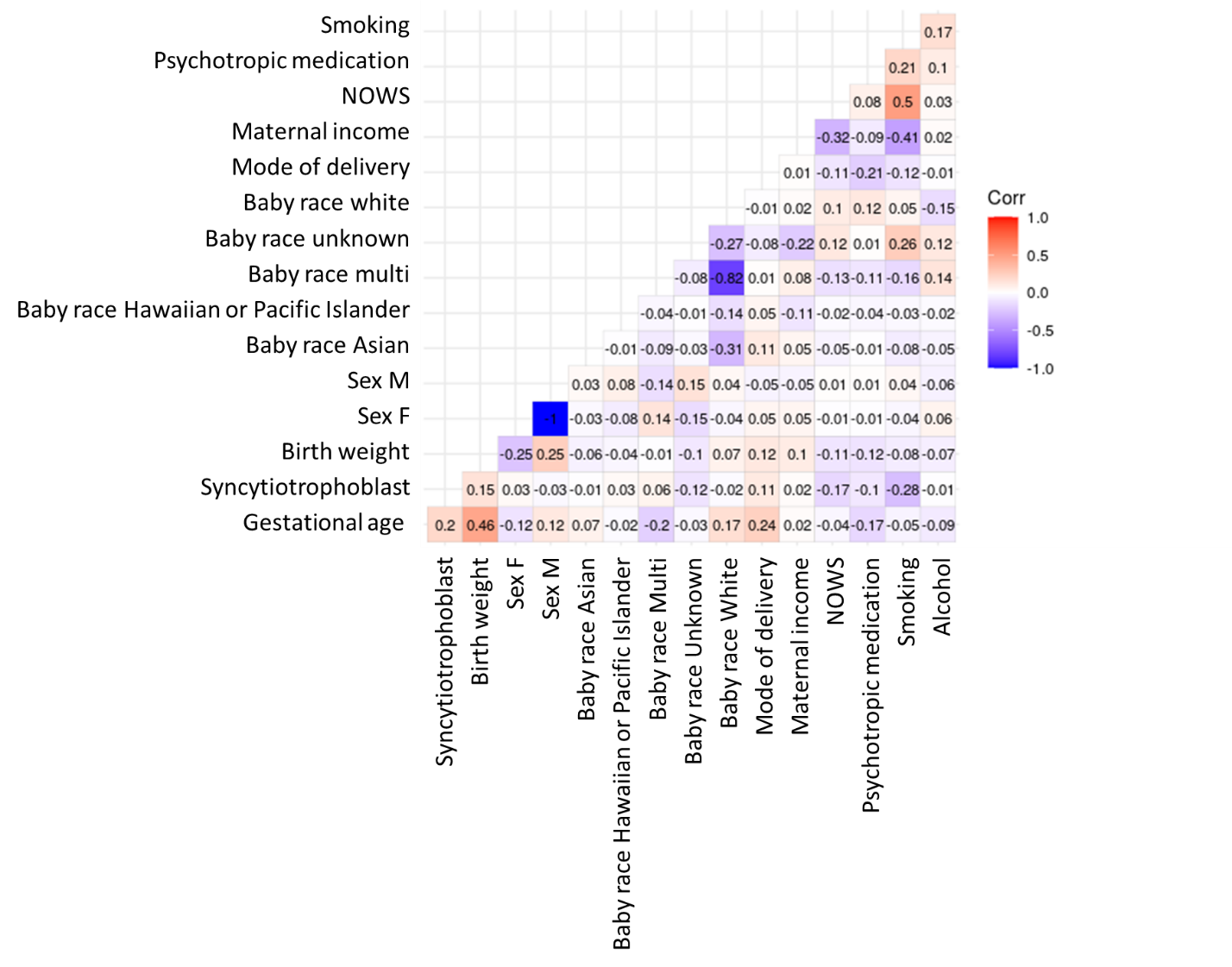
Supplementary Figure 1. Correlations between demographical variables and DNA methylation data.** A) Pearson’s r are reported for every pairwise comparison. Blue colours indicate negative correlations, whereas red colours indicate positive correlations. B) Correlation between demographical variables are being shown for the top nine principal components (PC) derived from the DNA methylation data. The first PC explains 6% of variance. The coloured boxes indicate the p-value of the association between covariates and DNA methylation data.

**B**

**A**


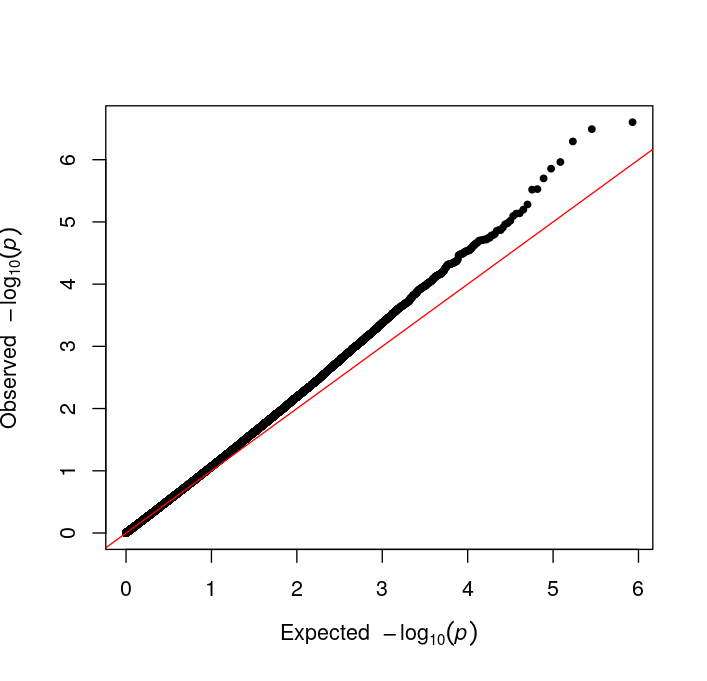


**Supplementary Figure 2.** **Quantile-quantile plot for epigenome-wide association study of in utero opioid exposure.** Each point represents a single CpG site of one CMP. The x-axis reflects the expected −log_10_(*p*-values), and the y-axis reflects the observed −log_10_(*p*-values).


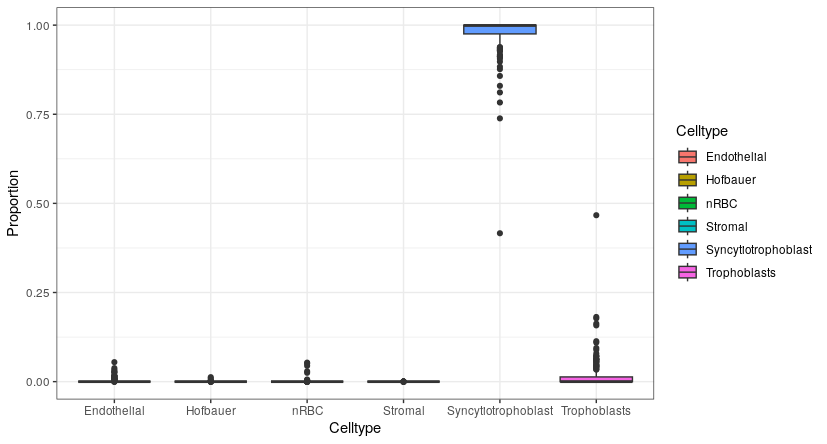


**Supplementary Figure 3. Estimated placental cell type proportions.** Cell type proportions were estimated based on DNA methylation levels through epigenomic deconvolution with the planet R package.


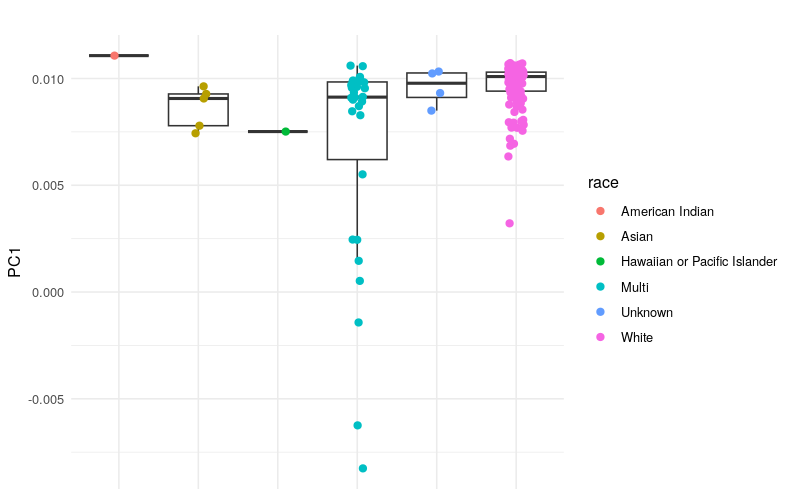

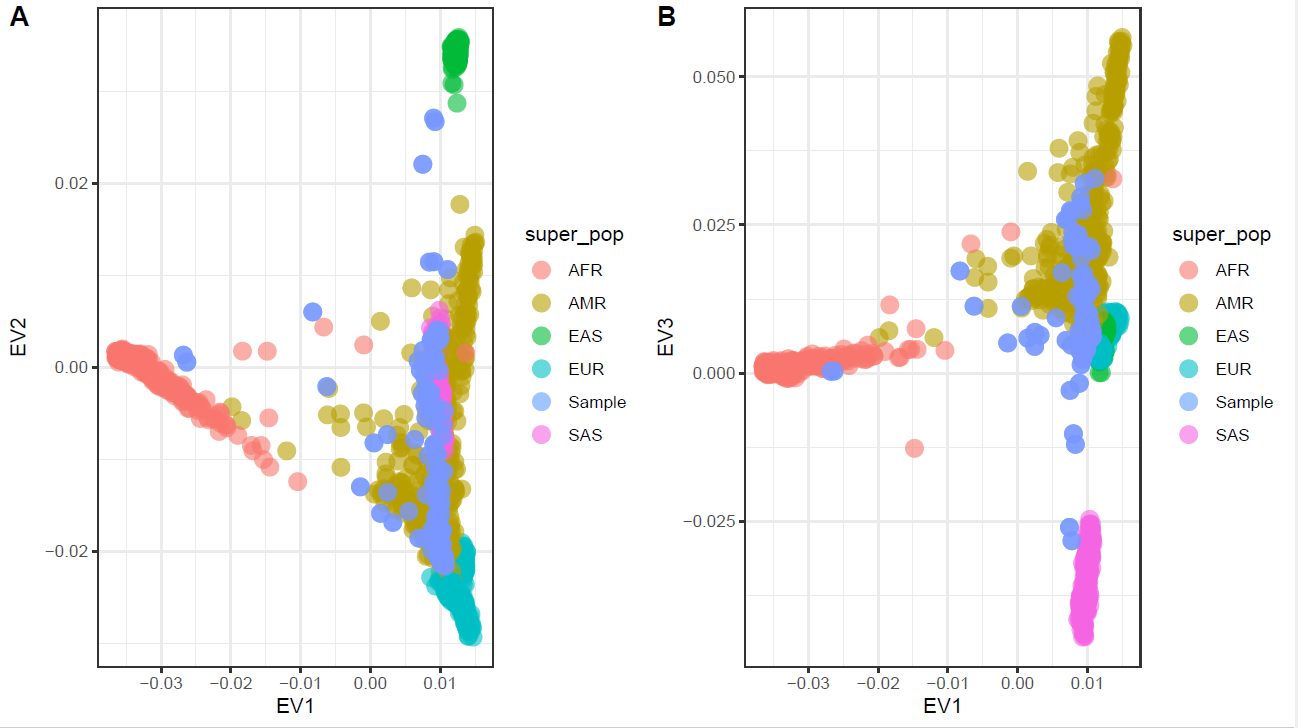


**D**

**C**


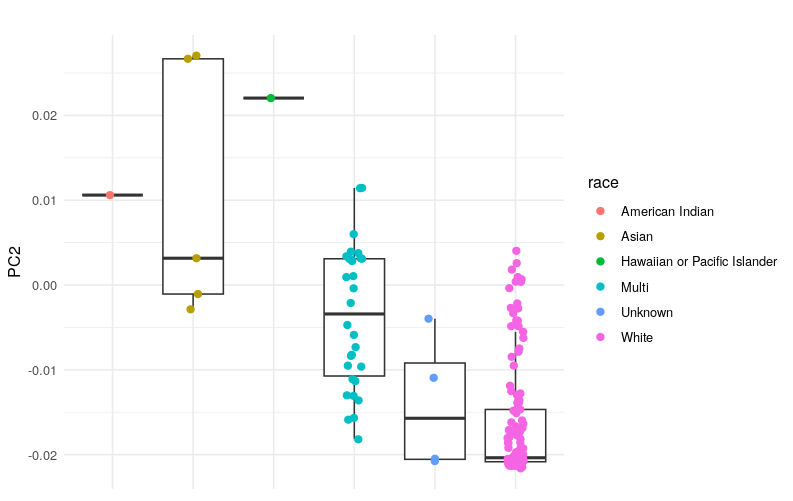


**Supplementary Figure 4. Principal component analysis of genetic ancestry data.** Cleaned genotyping data were combined with 2504 samples from the 1000 Genomes Project (1KGP) with multiple ancestries. PCA was performed on all 2667 samples, A and B) and the first two genetic PCs were selected as covariates reflecting genetic ancestry. AFR = African; AMR = American; EAS = East Asian; EUR = European; EV = Eigenvector; Sample = samples in current project; SAS = South Asian. C and D) The first two genetic PCs were different among the mother-reported infant’s race.

**Supplementary Table 1. Look-up of SNP Association in GWASs for Substance Use**

| **Substance use disorder outcome** | **Sample size GWAS** | **Reference** | ***p*-value** | **Effect size (effect allele)** |
| --- | --- | --- | --- | --- |
| **Alcohol dependence** | Case = 14,904,  Control = 37,944 | 10.1038/s41593-018-0275-1 | NA | NA |
| **Alcohol use disorder test** | 141,932 | 10.1176/appi.ajp.2018.18040369 | 0.1157897 | 0.0051720 (G) |
| **Ongoing addiction to alcohol** | Case = 1184, Control = 1555 | http://www.nealelab.is/uk-biobank/ | 0.913779 | −0.004165540 (A) |
| **Drinks per week** | 666,978 | 10.1038/s41586-022-05477-4 | 0.359075 | 0.004830 (A) |
| **Nicotine dependence** | 58,000 | 10.1038/s41467-020-19265-z | 0.5532 | −0.0177 (A) |
| **Age of initiation of regular smoking** | 323,386 | 10.1038/s41586-022-05477-4 | 0.889000 | 0.00107 (A) |
| **Cigarettes per day** | 326,497 | 10.1038/s41586-022-05477-4 | 0.0930 | 0.01290 (A) |
| **Smoking initiation** | 804,585 | 10.1038/s41586-022-05477-4 | 0.999254 | −0.000004 (A) |
| **Smoking cessation** | 388,313 | 10.1038/s41586-022-05477-4 | 0.857 | 0.00125 (A) |
| **Current tobacco smoker** | 360,797 | http://www.nealelab.is/uk-biobank/ | 0.505675 | −0.002315940 (A) |
| **Opioid dependence** | Case = 4503, Control = 4173  (exposed) | 10.1038/s41380-020-0677-9 | NA | NA |
| **Opioid exposure** | Case = 4173, Control = 32,500 | 10.1038/s41380-020-0677-9 | NA | NA |
| **Cannabis use disorder** | Case = 20,916, Control = 363,116 | [10.1016/S2215-0366(20)30339-4](https://doi.org/10.1016/S2215-0366(20)30339-4) | 0.5079 | −0.662 (A)  *Z-scores* |
| **Ongoing addiction or dependency to over-the-counter medication** | Case = 420, Control = 557 | http://www.nealelab.is/uk-biobank/ | 0.628999 | −0.0339574 (A) |

**Supplementary Table 2. mQTL SNP Distributions in Opioid-Exposed Infants With and Without NOWS**

| **SNP** | **Genotype** | | | ***p*-value** |
| --- | --- | --- | --- | --- |
| **rs117304669** |  |  |  | 0.444 |
| NOWS | 1 | 1 | 9 |  |
| No NOWS | 0 | 0 | 8 |  |
